# Supplementary material for: The Small RNA Universe of Capitella teleta
Source: Front Mol Biosci. 2022 Feb 25;9:802814. doi: 10.3389/fmolb.2022.802814 (PMC8915122; doi:10.3389/fmolb.2022.802814)
Supplement: Supplementary file 1 [file DataSheet1.ZIP › Supplement/known/CAPTEscaffold_135_11086.pdf]

Provisional ID	: CAPTEscaffold_135_11086
Score total	: 1820151.9
Score for star read(s)	: 3.9
Score for read counts	: 1820141.6
Score for mfe	: 1.9
Score for randfold	: 1.6
Score for cons. seed	: 3
Total read count	: 3570141
Mature read count	: 3570100
Loop read count	: 0
Star read count	: 41

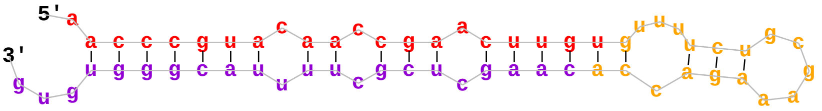

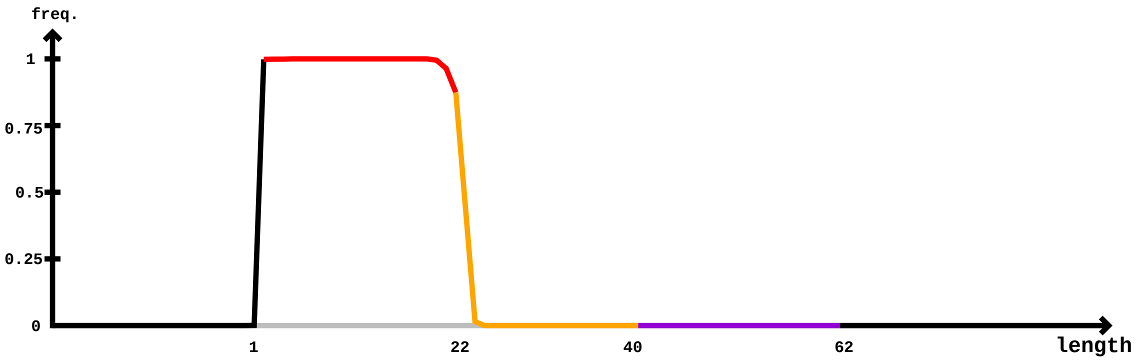

Mature **Star**

5'-	aaugucugccaaguagcgggaacccguacaaccgaacuuguguuuucugcgaaagaccacaagcucgcuuuuacgggugugcguugcugugggccucucgguccguga	-3'	obs	
	aaugucugccaaguagcgggaacccguacaaccgaacuuguguuuucugcgaaagaccacaagcucgcuuuuacgggugugcguugcugugggccucucgguccguga		exp	
	aaugucugccaaguagcgggaacccguacaaccgaacuuguguuuucugcgaaagaccacaagcucgcuuuuacgggugugcguugcugugggccucucgguccguga		known	
	...........(((((((.(.(((((((.((.(((.((((((...(((.....))).)))))).))).)).))))))).).)))))))..(((((....)))))....	reads	mm	sample
	aaugucugccaaguagcggg..	9	0	seq
	..................ggaacccguacaaccgaacuug..	20	0	seq
	..................ggaacccguacaaccgaacuugu...	60	0	seq
	..................ggaacccguacaaccAaacuugu...	1	1	seq
	..................ggaacccguacaaccgaacuugug..	1	0	seq
	...................gaacccguacaaccgaacu..	1	0	seq
	...................Caacccguacaaccgaacu..	5	1	seq
	...................gaacccguacaaccgaacuu...	11	0	seq
	...................Caacccguacaaccgaacuu...	34	1	seq
	...................Uaacccguacaaccgaacuug..	1	1	seq
	...................gaacccguacaacUgaacuug..	1	1	seq
	...................gaacccguacaaccgaacuug..	34	0	seq
	...................Caacccguacaaccgaacuug..	143	1	seq
	...................Aaacccguacaaccgaacuugu...	5	1	seq
	...................gaacccguacaaccAaacuugu...	1	1	seq
	...................gaCcccguacaaccgaacuugu...	1	1	seq
	...................gaacccguacaaccgaacuugu...	515	0	seq
	...................gaacccguacaacUgaacuugu...	1	1	seq
	...................Uaacccguacaaccgaacuugu...	1	1	seq
	...................gaacccguacaaccgaacuAgu...	1	1	seq
	...................gaacccgCacaaccgaacuugu...	1	1	seq
	...................gaacUcguacaaccgaacuugu...	1	1	seq
	...................gaaAccguacaaccgaacuugu...	1	1	seq
	...................gaGcccguacaaccgaacuugu...	1	1	seq
	...................Caacccguacaaccgaacuugu...	581	1	seq
	...................Caacccguacaaccgaacuugug..	76	1	seq
	...................Aaacccguacaaccgaacuugug..	7	1	seq
	...................gaacccguacaaccgaacuugug..	15	0	seq
	...................gaacccguacaaccgaacuugugA...	3	1	seq
	...................gaacccguacaaccgaacuugugAu..	1	1	seq
	....................aacccguacaaAcgaacu..	15	1	seq
	....................aacccguacaaccgaacG..	8	1	seq

```
                    Mature                  Star
aaugucugccaaguagcgggaacccguacaaccgaacuuguguuuucugcgaaagaccacaagcucgcuuuuacggguguggcguugcugugggccucucgguccguga
....................aacccUuacaaccgaacu......................................................................   2       1   seq
....................aacccguacaGccgaacu......................................................................   5       1   seq
....................Nacccguacaaccgaacu......................................................................   6       1   seq
....................aacccguacaaccgaaAu......................................................................   1       1   seq
....................Cacccguacaaccgaacu......................................................................   1       1   seq
....................aacccguacaaccgaacu......................................................................   17839   0   seq
....................aacccguacUaccgaacu......................................................................   1       1   seq
....................aNcccguacaaccgaacu......................................................................   3       1   seq
....................aacccguacaaccgUacu......................................................................   3       1   seq
....................aacccguacaUccgaacu......................................................................   2       1   seq
....................aacccguacaaccgaaUu......................................................................   2       1   seq
....................aaAccguacaaccgaacu......................................................................   6       1   seq
....................aacccguacGaccgaacu......................................................................   5       1   seq
....................aacAcguacaaccgaacu......................................................................   2       1   seq
....................aacccguacaacGgaacu......................................................................   2       1   seq
....................aacccguaAaaccgaacu......................................................................   1       1   seq
....................aacccguacaacAgaacu......................................................................   11      1   seq
....................aacccguacaacUgaacu......................................................................   8       1   seq
....................Gacccguacaaccgaacu......................................................................   14      1   seq
....................aacccguGcaaccgaacu......................................................................   3       1   seq
....................aacccguUcaaccgaacu......................................................................   1       1   seq
....................aCcccguacaaccgaacu......................................................................   1       1   seq
....................aaccAguacaaccgaacu......................................................................   8       1   seq
....................aacccguacaaccgGacu......................................................................   3       1   seq
....................aaccGguacaaccgaacu......................................................................   1       1   seq
....................aacccgCacaaccgaacu......................................................................   1       1   seq
....................aacccguacaaccgaacC......................................................................   4       1   seq
....................aGcccguacaaccgaacu......................................................................   5       1   seq
....................aacccCuacaaccgaacu......................................................................   10      1   seq
....................aacccguacaaccUaacu......................................................................   2       1   seq
....................aacccAuacaaccgaacu......................................................................   4       1   seq
....................aUcccguacaaccgaacu......................................................................   1       1   seq
....................aaUccguacaaccgaacu......................................................................   3       1   seq
....................aacccguacaaUcgaacu......................................................................   1       1   seq
....................Uacccguacaaccgaacu......................................................................   3       1   seq
....................aacccguacaaccgaacA......................................................................   10      1   seq
....................aacccguacaaccCaacu......................................................................   2       1   seq
....................aacccguacaaccAaacu......................................................................   64      1   seq
....................aacccguacaaccgaUcu......................................................................   1       1   seq
....................aacUcguacaaccgaacu......................................................................   7       1   seq
....................aacccguaUaaccgaacu......................................................................   1       1   seq
....................aacccgGacaaccgaacu......................................................................   3       1   seq
....................aacccguacaaccgaGcu......................................................................   7       1   seq
....................aacccgAacaaccgaacu......................................................................   20      1   seq
....................aaccUguacaaccgaacu......................................................................   14      1   seq
....................aacccguacaaccgGacuu.....................................................................   18      1   seq
....................aaccAguacaaccgaacuu.....................................................................   32      1   seq
....................aacccguacaaccgaaAuu.....................................................................   12      1   seq
....................aacccguacaaccUaacuu.....................................................................   8       1   seq
....................aacccguacaaccgaacuC.....................................................................   9       1   seq
....................aacccguacaaUcgaacuu.....................................................................   39      1   seq
....................aaGccguacaaccgaacuu.....................................................................   1       1   seq
....................aGcccguacaaccgaacuu.....................................................................   48      1   seq
....................aacccguaAaaccgaacuu.....................................................................   9       1   seq
....................aacccgCacaaccgaacuu.....................................................................   20      1   seq
....................aacccAuacaaccgaacuu.....................................................................   38      1   seq
....................Nacccguacaaccgaacuu.....................................................................   27      1   seq
....................aNcccguacaaccgaacuu.....................................................................   4       1   seq
....................Cacccguacaaccgaacuu.....................................................................   11      1   seq
....................aacccguacaacUgaacuu.....................................................................   98      1   seq
....................aacccguacaaccgaaGuu.....................................................................   3       1   seq
....................aacccguacaaccgaacCu.....................................................................   24      1   seq
....................aacccguacaaccgaacuu.....................................................................   109035  0   seq
....................aacccguacaCccgaacuu.....................................................................   2       1   seq
....................aacccguacaUccgaacuu.....................................................................   10      1   seq
....................aacccguacaaccgaGcuu.....................................................................   30      1   seq
....................aacccguacaaccgaUcuu.....................................................................   9       1   seq
....................aacccguacaaccgUacuu.....................................................................   2       1   seq
....................aacccgAacaaccgaacuu.....................................................................   165     1   seq
....................aacccguacaacGgaacuu.....................................................................   9       1   seq
```

Mature Star

```
aaugucugccaaguagcgggaacccguacaaccgaacuuguguuuucugcgaaagaccacaagcucgcuuuuacgggugugcguugcugugggccucucgguccguga
....................aacccguacaaccgaacGu.....................................................................	5	1	seq
....................aacccUuacaaccgaacuu.....................................................................	6	1	seq
....................aacccgGacaaccgaacuu.....................................................................	16	1	seq
....................aacccguacaaGcgaacuu.....................................................................	2	1	seq
....................aacccguacaaccgaacuG.....................................................................	33	1	seq
....................aacccguUcaaccgaacuu.....................................................................	12	1	seq
....................aacccguacaaccgCacuu.....................................................................	1	1	seq
....................aacccguCcaaccgaacuu.....................................................................	1	1	seq
....................aUcccguacaaccgaacuu.....................................................................	6	1	seq
....................Gacccguacaaccgaacuu.....................................................................	79	1	seq
....................aacccguacGaccgaacuu.....................................................................	31	1	seq
....................aacccguacCaccgaacuu.....................................................................	2	1	seq
....................aaccGguacaaccgaacuu.....................................................................	7	1	seq
....................aacUcguacaaccgaacuu.....................................................................	47	1	seq
....................aaUccguacaaccgaacuu.....................................................................	8	1	seq
....................aacccguGcaaccgaacuu.....................................................................	15	1	seq
....................aacccguaUaaccgaacuu.....................................................................	9	1	seq
....................aacccguacaaccgaCcuu.....................................................................	1	1	seq
....................aacccguacaacAgaacuu.....................................................................	80	1	seq
....................aacccguacaGccgaacuu.....................................................................	11	1	seq
....................aacccguaGaaccgaacuu.....................................................................	6	1	seq
....................aaAccguacaaccgaacuu.....................................................................	32	1	seq
....................aacccguacUaccgaacuu.....................................................................	8	1	seq
....................aacccguacaaccgaaUuu.....................................................................	15	1	seq
....................aacccguacaaccAaacuu.....................................................................	508	1	seq
....................aacccguacaaccCaacuu.....................................................................	11	1	seq
....................Uacccguacaaccgaacuu.....................................................................	3	1	seq
....................aaccUguacaaccgaacuu.....................................................................	81	1	seq
....................aacAcguacaaccgaacuu.....................................................................	18	1	seq
....................aacccguacaaAcgaacuu.....................................................................	91	1	seq
....................aacGcguacaaccgaacuu.....................................................................	2	1	seq
....................aacccguacaaccgaacuA.....................................................................	74	1	seq
....................aacccguacaaccgaacAu.....................................................................	73	1	seq
....................aacccCuacaaccgaacuu.....................................................................	7	1	seq
....................Naccccguacaaccgaacuug....................................................................	81	1	seq
....................aCcccguacaaccgaacuug....................................................................	5	1	seq
....................Uacccguacaaccgaacuug....................................................................	11	1	seq
....................aacccguacaaccgaacuAg....................................................................	192	1	seq
....................aacccguacaaccgaaUuug....................................................................	29	1	seq
....................aUcccguacaaccgaacuug....................................................................	22	1	seq
....................Gacccguacaaccgaacuug....................................................................	288	1	seq
....................aacccguacaaccgaacuuU....................................................................	42	1	seq
....................aacccguacaUccgaacuug....................................................................	32	1	seq
....................aacccguacaaccgaacuuC....................................................................	42	1	seq
....................aacccguacaaUcgaacuug....................................................................	41	1	seq
....................aacccguGcaaccgaacuug....................................................................	45	1	seq
....................aacccAuacaaccgaacuug....................................................................	85	1	seq
....................aacccguaUaaccgaacuug....................................................................	46	1	seq
....................aacccguacaaccCaacuug....................................................................	22	1	seq
....................aacccguaGaaccgaacuug....................................................................	17	1	seq
....................aacccguacaaccgaacuGg....................................................................	151	1	seq
....................aacccguacaaccgUacuug....................................................................	6	1	seq
....................aacccguacaaccgaacuug....................................................................	314098	0	seq
....................aacccUuacaaccgaacuug....................................................................	29	1	seq
....................aacccguacaaGcgaacuug....................................................................	9	1	seq
....................aacccguacaaccgCacuug....................................................................	5	1	seq
....................aacUcguacaaccgaacuug....................................................................	108	1	seq
....................aacccNuacaaccgaacuug....................................................................	2	1	seq
....................Cacccguacaaccgaacuug....................................................................	25	1	seq
....................aacccguacaCccgaacuug....................................................................	9	1	seq
....................aaNccguacaaccgaacuug....................................................................	3	1	seq
....................aaccUguacaaccgaacuug....................................................................	191	1	seq
....................aacAcguacaaccgaacuug....................................................................	46	1	seq
....................aacccCuacaaccgaacuug....................................................................	37	1	seq
....................aacccgGacaaccgaacuug....................................................................	36	1	seq
....................aacccguacaaccgaaAuug....................................................................	50	1	seq
....................aacccguacaacGgaacuug....................................................................	31	1	seq
....................aacccguacaaccgaacAug....................................................................	212	1	seq
....................aacccguUcaaccgaacuug....................................................................	39	1	seq
....................aacccguacaaccgaacuCg....................................................................	41	1	seq
```

```
                    Mature                                 Star
aaugucugccaaguagcgggaacccguacaaccgaacuuguguuuucugcgaaagaccacaagcucgcuuuuacggguguguguugcugugggccucucgguccguga
....................aacccguacaaccgaacCug....................................................................  33     1  seq
....................aNcccguacaaccgaacuug....................................................................  16     1  seq
....................aacccguacaacAgaacuug....................................................................  214    1  seq
....................aacccguacaaccgaacGug....................................................................  25     1  seq
....................aaccAguacaaccgaacuug....................................................................  102    1  seq
....................aacccguaAaaccgaacuug....................................................................  57     1  seq
....................aacccguacaaAcgaacuug....................................................................  192    1  seq
....................aacccguacaaccgaCcuug....................................................................  4      1  seq
....................aacccgAacaaccgaacuug....................................................................  398    1  seq
....................aacccguacaaccAaacuug....................................................................  1466   1  seq
....................aaAccguacaaccgaacuug....................................................................  122    1  seq
....................aacccguacaaccgaGcuug....................................................................  131    1  seq
....................aacccgCacaaccgaacuug....................................................................  37     1  seq
....................aacccguacaaccgaacuuA....................................................................  53     1  seq
....................aacccguacaaccgaUcuug....................................................................  20     1  seq
....................aaGccguacaaccgaacuug....................................................................  6      1  seq
....................aacccguacUaccgaacuug....................................................................  23     1  seq
....................aacccguCcaaccgaacuug....................................................................  1      1  seq
....................aaccGguacaaccgaacuug....................................................................  8      1  seq
....................aGcccguacaaccgaacuug....................................................................  183    1  seq
....................aacccguacaaccgGacuug....................................................................  58     1  seq
....................aacccguacGaccgaacuug....................................................................  66     1  seq
....................aacccguacaaccUaacuug....................................................................  36     1  seq
....................aacccguacaacUgaacuug....................................................................  252    1  seq
....................aacccguacNaccgaacuug....................................................................  2      1  seq
....................aacccguacCaccgaacuug....................................................................  2      1  seq
....................aacGcguacaaccgaacuug....................................................................  11     1  seq
....................aaUccguacaaccgaacuug....................................................................  19     1  seq
....................aacccguacaaccgaaGuug....................................................................  16     1  seq
....................aacccguacaGccgaacuug....................................................................  55     1  seq
....................Cacccguacaaccgaacuugu...................................................................  286    1  seq
....................aacccguacaaccgaacuCgu...................................................................  328    1  seq
....................aacccguacaaccgGacuugu...................................................................  1447   1  seq
....................aacccguacaaccgaNcuugu...................................................................  3      1  seq
....................aacccguaUaaccgaacuugu...................................................................  396    1  seq
....................aacccguacaaccgaacCugu...................................................................  367    1  seq
....................aCcccguacaaccgaacuugu...................................................................  62     1  seq
....................Uacccguacaaccgaacuugu...................................................................  106    1  seq
....................aacUcguacaaccgaacuugu...................................................................  1112   1  seq
....................aacccguacaaccgaacuuCu...................................................................  345    1  seq
....................aacccguacUaccgaacuugu...................................................................  180    1  seq
....................aacAcguacaaccgaacuugu...................................................................  514    1  seq
....................aacccguacaaAcgaacuugu...................................................................  1802   1  seq
....................aacccguaGaaccgaacuugu...................................................................  133    1  seq
....................aacccguacaaGcgaacuugu...................................................................  63     1  seq
....................aaccGguacaaccgaacuugu...................................................................  135    1  seq
....................aacccguacaaccgaacuugG...................................................................  2987   1  seq
....................aacccguacaGccgaacuugu...................................................................  523    1  seq
....................aacccguacaaccgUacuugu...................................................................  156    1  seq
....................aacccguacaaccgaacuugN...................................................................  4      1  seq
....................aacccguacaCccgaacuugu...................................................................  62     1  seq
....................aacccguacaaccgaacuuNu...................................................................  1      1  seq
....................aacccguacaacUgaacuugu...................................................................  1608   1  seq
....................Gacccguacaaccgaacuugu...................................................................  1827   1  seq
....................aNcccguacaaccgaacuugu...................................................................  121    1  seq
....................aacccguacaaccgaaUuugu...................................................................  339    1  seq
....................aaAccguacaaccgaacuugu...................................................................  983    1  seq
....................aacccguacaaccgaacuuUu...................................................................  213    1  seq
....................aaccAguacaaccgaacuugu...................................................................  938    1  seq
....................aacccguacGaccgaacuugu...................................................................  542    1  seq
....................aacccguacaaccgaacAugu...................................................................  1582   1  seq
....................aacccguacaaccgaGcuugu...................................................................  948    1  seq
....................aacccguacaaccgaacuAgu...................................................................  1449   1  seq
....................aacccgNacaaccgaacuugu...................................................................  2      1  seq
....................aacccguacaUccgaacuugu...................................................................  354    1  seq
....................aacccguacaaccUaacuugu...................................................................  339    1  seq
....................aacccguacaaccgaUcuugu...................................................................  221    1  seq
....................aacccguacaaccgaacuuAu...................................................................  281    1  seq
....................aacccguacaaccgaaGuugu...................................................................  93     1  seq
....................aacccguacaaccAaacuugu...................................................................  13139  1  seq
```

```
                    Mature                                 Star
aaugucugccaaguagcgggaacccguacaaccgaacuuguguuuucugcgaaagaccacaagcucgcuuuuacgggugugcguugcugugggccucucgguccguga
....................aaGccguacaaccgaacuugu...................................................................    85       1    seq
....................aacccguacaaccgaacuGgu...................................................................    159      1    seq
....................aacccguacCaccgaacuugu...................................................................    26       1    seq
....................aacccguacaaccCaacuugu...................................................................    247      1    seq
....................aacccguacaaccgaacuNgu...................................................................    3        1    seq
....................aacccCuacaaccgaacuugu...................................................................    324      1    seq
....................aacccguacaaUcgaacuugu...................................................................    379      1    seq
....................aacccUuacaaccgaacuugu...................................................................    284      1    seq
....................aacccguacaaccgaacGugu...................................................................    181      1    seq
....................aacccguacaaccgCacuugu...................................................................    40       1    seq
....................aacccguacaaccgaacuugC...................................................................    306      1    seq
....................aaccUguacaaccgaacuugu...................................................................    2062     1    seq
....................aacccguacaaccgaaNuugu...................................................................    4        1    seq
....................aacccgCacaaccgaacuugu...................................................................    507      1    seq
....................aacccguacaaccgaCcuugu...................................................................    27       1    seq
....................aacccguacNaccgaacuugu...................................................................    7        1    seq
....................aacccguacaNccgaacuugu...................................................................    1        1    seq
....................aUcccguacaaccgaacuugu...................................................................    167      1    seq
....................aacccguGcaaccgaacuugu...................................................................    402      1    seq
....................aacccgGacaaccgaacuugu...................................................................    234      1    seq
....................aacccAuacaaccgaacuugu...................................................................    675      1    seq
....................aacccguCcaaccgaacuugu...................................................................    34       1    seq
....................aacccguacaacAgaacuugu...................................................................    1970     1    seq
....................aaNccguacaaccgaacuugu...................................................................    8        1    seq
....................aacGcguacaaccgaacuugu...................................................................    80       1    seq
....................aacccguacaacGgaacuugu...................................................................    211      1    seq
....................aacccgAacaaccgaacuugu...................................................................    3220     1    seq
....................aacccguacaaccgaacuugu...................................................................    2919746  0    seq
....................aacccguaAaaccgaacuugu...................................................................    504      1    seq
....................aacccguUcaaccgaacuugu...................................................................    431      1    seq
....................Nacccguacaaccgaacuugu...................................................................    709      1    seq
....................aacccguacaaccgaacuugA...................................................................    2439     1    seq
....................aacccguacaaccNaacuugu...................................................................    1        1    seq
....................aGcccguacaaccgaacuugu...................................................................    1899     1    seq
....................aacccguacaaccgaaAuugu...................................................................    420      1    seq
....................aaUccguacaaccgaacuugu...................................................................    250      1    seq
....................aacccNuacaaccgaacuugu...................................................................    15       1    seq
....................aacccguacaaccgaUcuugug..................................................................    8        1    seq
....................aacccCuacaaccgaacuugug..................................................................    9        1    seq
....................aacccguacaaccgaacuuguU..................................................................    964      1    seq
....................aacccguacaaccgaacuGgug..................................................................    5        1    seq
....................aaccUguacaaccgaacuugug..................................................................    65       1    seq
....................aNcccguacaaccgaacuugug..................................................................    2        1    seq
....................aacccguacaaccgGacuugug..................................................................    58       1    seq
....................aacccguacaaccgaacuuguC..................................................................    11       1    seq
....................aaccAguacaaccgaacuugug..................................................................    24       1    seq
....................aacAcguacaaccgaacuugug..................................................................    9        1    seq
....................aaUccguacaaccgaacuugug..................................................................    15       1    seq
....................aacccguacaaccgaacCugug..................................................................    10       1    seq
....................Nacccguacaaccgaacuugug..................................................................    23       1    seq
....................aacccguacaaccgaacuuguA..................................................................    717      1    seq
....................aacccguacaaccgaacuugAg..................................................................    76       1    seq
....................aacccguacaaccgaacuugGg..................................................................    14       1    seq
....................aacccguacaaccgaacuugug..................................................................    83217    0    seq
....................aUcccguacaaccgaacuugug..................................................................    4        1    seq
....................aacccguGcaaccgaacuugug..................................................................    11       1    seq
....................aacccguUcaaccgaacuugug..................................................................    13       1    seq
....................aacccguacaaccgUacuugug..................................................................    2        1    seq
....................aGcccguacaaccgaacuugug..................................................................    144      1    seq
....................aacccguacaaccgaacAugug..................................................................    54       1    seq
....................aacccguacaUccgaacuugug..................................................................    10       1    seq
....................aacccgGacaaccgaacuugug..................................................................    4        1    seq
....................aacccgAacaaccgaacuugug..................................................................    86       1    seq
....................aacUcguacaaccgaacuugug..................................................................    47       1    seq
....................aacccguacaaUcgaacuugug..................................................................    8        1    seq
....................aaAccguacaaccgaacuugug..................................................................    25       1    seq
....................aacccguacaacGgaacuugug..................................................................    2        1    seq
....................aacccguacaacAgaacuugug..................................................................    61       1    seq
....................aacccguacaGccgaacuugug..................................................................    14       1    seq
....................aacccguaAaaccgaacuugug..................................................................    20       1    seq
```

Mature Star

```
aaugucugccaaguagcgggaacccguacaaccgaacuuguguuuucugcgaaagaccacaagcucgcuuuuacggguguggcguugcugugggccucucgguccguga
....................aacccguacaaccgaaAuugug..................................................................  11     1  seq
....................aacccguacaaAcgaacuugug..................................................................  59     1  seq
....................aacccguaUaaccgaacuugug..................................................................  11     1  seq
....................Cacccguacaaccgaacuugug..................................................................  11     1  seq
....................aacccguacaaccgaaUuugug..................................................................  26     1  seq
....................aacccUuacaaccgaacuugug..................................................................  8      1  seq
....................aacccNuacaaccgaacuugug..................................................................  1      1  seq
....................aacccAuacaaccgaacuugug..................................................................  15     1  seq
....................aacccguacaaccgaacuugCg..................................................................  6      1  seq
....................aacccgCacaaccgaacuugug..................................................................  14     1  seq
....................aacccguacaaccgaacuCgug..................................................................  18     1  seq
....................aacccguacaaccgaacuuCug..................................................................  8      1  seq
....................aacccguaGaaccgaacuugug..................................................................  5      1  seq
....................Uacccguacaaccgaacuugug..................................................................  4      1  seq
....................aacccguacaaccgaacuAgug..................................................................  47     1  seq
....................Gacccguacaaccgaacuugug..................................................................  48     1  seq
....................aaGccguacaaccgaacuugug..................................................................  3      1  seq
....................aaccGguacaaccgaacuugug..................................................................  2      1  seq
....................aacccguacaaccAaacuugug..................................................................  443    1  seq
....................aacccguacaaccUaacuugug..................................................................  17     1  seq
....................aacccguacGaccgaacuugug..................................................................  20     1  seq
....................aacccguacaaccCaacuugug..................................................................  4      1  seq
....................aacccguacaaccgaacGugug..................................................................  6      1  seq
....................aacccguacaaccgaGcuugug..................................................................  80     1  seq
....................aacccguacaaccgaacuuAug..................................................................  11     1  seq
....................aacccguacUaccgaacuugug..................................................................  5      1  seq
....................aacccguacaacUgaacuugug..................................................................  58     1  seq
....................aacccguacaaccgaacuuUug..................................................................  2      1  seq
....................aacccguacaaccgaacuAgugu.................................................................  1      1  seq
....................aacccguUcaaccgaacuugugu.................................................................  1      1  seq
....................aacccguaAaaccgaacuugugu.................................................................  2      1  seq
....................aacccguacaaccgaacuugugu.................................................................  2941   0  seq
....................aaccAguacaaccgaacuugugu.................................................................  1      1  seq
....................aacccguacaaccgaGcuugugu.................................................................  4      1  seq
....................Cacccguacaaccgaacuugugu.................................................................  1      1  seq
....................aacccguacaaccgaacuuUugu.................................................................  1      1  seq
....................aacccguacaaccgaacAugugu.................................................................  1      1  seq
....................aacccguacaaccgaaUuugugu.................................................................  2      1  seq
....................aaccUguacaaccgaacuugugu.................................................................  2      1  seq
....................aacccgAacaaccgaacuugugu.................................................................  3      1  seq
....................aacccguacaaccgaacuugugC.................................................................  31     1  seq
....................aacccUuacaaccgaacuugugu.................................................................  1      1  seq
....................Gacccguacaaccgaacuugugu.................................................................  1      1  seq
....................aacccguacaaccgaacuuguCu.................................................................  2      1  seq
....................aacccguacaaccgaacuugugG.................................................................  6317   1  seq
....................aacccguacaaccgaacuuguUu.................................................................  11     1  seq
....................aacccguacaaccgaacuugugN.................................................................  1      1  seq
....................aacccguacaaccgaacuugAgu.................................................................  1      1  seq
....................aacccguacaacAgaacuugugu.................................................................  3      1  seq
....................aacccgGacaaccgaacuugugu.................................................................  1      1  seq
....................aaAccguacaaccgaacuugugu.................................................................  1      1  seq
....................aUcccguacaaccgaacuugugu.................................................................  2      1  seq
....................aacccguacaaccAaacuugugu.................................................................  6      1  seq
....................aacccguacaaccgaacuugugA.................................................................  42931  1  seq
....................aacccguacaaAcgaacuugugu.................................................................  4      1  seq
....................aGcccguacaaccgaacuugugu.................................................................  3      1  seq
....................aacccguacaaccgaacuugugAu................................................................  244    1  seq
....................aacccguacaaccgaacuugugGu................................................................  2      1  seq
....................aacccguacaaccgaacuuguguC................................................................  13     1  seq
....................aacccguacaaccgaacuuguUuu................................................................  2      1  seq
....................aacccguacaaccgaacuuguguu................................................................  8      0  seq
....................aacccguacaaccgaacuuguguA................................................................  17     1  seq
....................aacccguacaaccgGacuuguguu................................................................  1      1  seq
....................aacccguacaaccgaacuuguguuu...............................................................  4      0  seq
....................aacccguacaaccgaacuuguguuA...............................................................  2      1  seq
....................aacccguacaaccgaacuuguguAu...............................................................  5      1  seq
....................aacccguacaaccgaacuuguUuuuu..............................................................  2      1  seq
.....................aAccguacaaccgaacuu.....................................................................  6      1  seq
.....................Gcccguacaaccgaacuu.....................................................................  6      1  seq
.....................acccguacaaccgaacuu.....................................................................  55     0  seq
```

Mature Star

```
aaugucugccaaguagcgggaacccguacaaccgaacuuguguuuucugcgaaagaccacaagcucgcuuuuacgggugugcguugcugugggccucucgguccguga
.....................acccguacaaccgaacAu.....................................................................	1	1	seq
.....................Ucccguacaaccgaacuug....................................................................	3	1	seq
.....................acccguacaaccgaacuuU....................................................................	1	1	seq
.....................acccguacaaccgaacAug....................................................................	4	1	seq
.....................acccguacaaccgaacuug....................................................................	142	0	seq
.....................aUccguacaaccgaacuug....................................................................	1	1	seq
.....................acccCuacaaccgaacuug....................................................................	2	1	seq
.....................Gcccguacaaccgaacuug....................................................................	19	1	seq
.....................aAccguacaaccgaacuug....................................................................	16	1	seq
.....................acccguacaaccgaacCugu...................................................................	1	1	seq
.....................acccguacaaccgaacuuUu...................................................................	2	1	seq
.....................acccguacaaccgaacuugG...................................................................	2	1	seq
.....................acccguacaaccAaacuugu...................................................................	5	1	seq
.....................acccguacaaccgaaGuugu...................................................................	1	1	seq
.....................acccCuacaaccgaacuugu...................................................................	21	1	seq
.....................Ucccguacaaccgaacuugu...................................................................	36	1	seq
.....................acccguacaaccgCacuugu...................................................................	2	1	seq
.....................Gcccguacaaccgaacuugu...................................................................	253	1	seq
.....................acccgAacaaccgaacuugu...................................................................	2	1	seq
.....................acccguacaaccgaacuugu...................................................................	1624	0	seq
.....................acccguacaaccgaacGugu...................................................................	1	1	seq
.....................aAccguacaaccgaacuugu...................................................................	126	1	seq
.....................acccguacaaccgaacuCgu...................................................................	1	1	seq
.....................acccguacaaccgaacAugu...................................................................	14	1	seq
.....................acccguacaaccCaacuugu...................................................................	2	1	seq
.....................acccguacaacUgaacuugu...................................................................	1	1	seq
.....................acccguaAaaccgaacuugu...................................................................	1	1	seq
.....................acccguacaaccgaacuuguU..................................................................	1	1	seq
.....................Gcccguacaaccgaacuugug..................................................................	12	1	seq
.....................acccguacaaccAaacuugug..................................................................	2	1	seq
.....................aAccguacaaccgaacuugug..................................................................	2	1	seq
.....................acccguacaaccgaacuugug..................................................................	94	0	seq
.....................acccguacaaccgaacuugugG.................................................................	1	1	seq
.....................acccguacaaccgaacuugugA.................................................................	14	1	seq
.....................acccguacaaccgaacuugugAu................................................................	1	1	seq
......................cccguacaaccgaacuug....................................................................	13	0	seq
......................cccguaAaaccgaacuug....................................................................	1	1	seq
......................cccguacaaccgaacuAgu...................................................................	1	1	seq
......................cccguacaaccAaacuugu...................................................................	2	1	seq
......................cUcguacaaccgaacuugu...................................................................	1	1	seq
......................cccguacaaccgaacuugu...................................................................	375	0	seq
......................ccAguacaaccgaacuugu...................................................................	1	1	seq
......................Accguacaaccgaacuugu...................................................................	2	1	seq
......................cccguacaacAgaacuugu...................................................................	1	1	seq
......................cccguacaaccgaacuugug..................................................................	14	0	seq
......................cccguacaaccgaacuugugA.................................................................	6	1	seq
......................cccguacaaccgaacuugugG.................................................................	1	1	seq
.......................Ucguacaaccgaacuugu...................................................................	4	1	seq
.......................ccguacaaccgaacuuAu...................................................................	1	1	seq
.......................ccAuacaaccgaacuugu...................................................................	1	1	seq
.......................cUguacaaccgaacuugu...................................................................	3	1	seq
.......................ccguacaaccAaacuugu...................................................................	17	1	seq
.......................cAguacaaccgaacuugu...................................................................	2	1	seq
.......................ccguacaaccgaacuugu...................................................................	3389	0	seq
.......................cNguacaaccgaacuugu...................................................................	1	1	seq
.......................ccguacaaccgaacuugG...................................................................	5	1	seq
.......................ccguGcaaccgaacuugu...................................................................	1	1	seq
.......................ccguacaaccgUacuugu...................................................................	1	1	seq
.......................Gcguacaaccgaacuugu...................................................................	1	1	seq
.......................ccguacaUccgaacuugu...................................................................	1	1	seq
.......................ccguacaacUgaacuugu...................................................................	2	1	seq
.......................ccgGacaaccgaacuugu...................................................................	1	1	seq
.......................ccguacaaccgaUcuugu...................................................................	1	1	seq
.......................ccguacaaGcgaacuugu...................................................................	1	1	seq
.......................Ncguacaaccgaacuugu...................................................................	1	1	seq
.......................ccguacaaccgaacuAgu...................................................................	4	1	seq
.......................ccguacaaccgaacAugu...................................................................	2	1	seq
.......................ccguacaaccgaaUuugu...................................................................	1	1	seq
.......................ccgAacaaccgaacuugu...................................................................	2	1	seq
.......................ccguacaacAgaacuugu...................................................................	3	1	seq
```

Mature **Star**

```
aaugucugccaaguagcgggaacccguacaaccgaacuuguguuuucugcgaaagaccacaagcucgcuuuuacgggugugcguugcugugggccucucgguccguga
.......................ccguacaacUgaacuugug..................................................................	1	1	seq
.......................ccguacaaccgaacuugug..................................................................	39	0	seq
.......................ccguacaaccgaacuuguU..................................................................	9	1	seq
.......................ccguacaaccgaacuugugG.................................................................	4	1	seq
.......................ccguacaaccgaacuugugA.................................................................	15	1	seq
.......................ccguacaaccgaacuugugu.................................................................	13	0	seq
........................cguacaaccgaacuuguU..................................................................	5	1	seq
........................cguacaaccgaacuugug..................................................................	10	0	seq
........................cguacaaccgaacuugugG.................................................................	2	1	seq
........................cguacaaccgaacuugugu.................................................................	12	0	seq
........................cguacaaccgaacuugugA.................................................................	7	1	seq
.........................guacaaccgaacuugugu.................................................................	2	0	seq
.......................................................accacaagcucgcuuuuacg.................................	2	0	seq
.......................................................accacaagcucgcuuuuacgg................................	2	0	seq
.......................................................accacaagcucgcuuuuacggg...............................	3	0	seq
...........................................................caagcucgcuuuuacggA...............................	1	1	seq
...........................................................caagcucgcuuuuacgggugu............................	4	0	seq
...........................................................caagcucgcuAuuacgggugug...........................	1	1	seq
...........................................................caaUcucgcuuuuacgggugug...........................	1	1	seq
...........................................................caagcucgcuuuuacgggugug...........................	32	0	seq
...........................................................caagcucgcuuuuacggguguG..........................	1	1	seq
...............................................................cucgcuuuuacgggugug...........................	1	0	seq
.................................................................................cguugcugugggccucucggu......	1	0	seq
```
